# CAR-T Cells Based on Novel BCMA Monoclonal Antibody Block Multiple Myeloma Cell Growth

**DOI:** 10.3390/cancers10090323

**Published:** 2018-09-11

**Authors:** Robert Berahovich, Hua Zhou, Shirley Xu, Yuehua Wei, Jasper Guan, Jian Guan, Hizkia Harto, Shuxiang Fu, Kaihuai Yang, Shuying Zhu, Le Li, Lijun Wu, Vita Golubovskaya

**Affiliations:** 1 ProMab Biotechnologies, 2600 Hilltop Drive, Richmond, CA 94806, USA; robert.berahovich@promab.com (R.B.); huazhou369@gmail.com (H.Z.); shirley.xu@promab.com (S.X.); yuehua.wei@promab.com (Y.W.); jasper.guan@promab.com (J.G.); jian.guan@promab.com (J.G.); hizkia.harto@promab.com (H.H.); simon.li@promab.com (L.L.); john@promab.com (L.W.); 2 Forevertek Biotechnology Co., Ltd., Building M0, Oversea Graduate Park National High-Tech Industrial Zone, Changsha 410003, China; promab8807@126.com (S.F.); yangkaihuai520@126.com (K.Y.); shuying256@163.com (S.Z.)

**Keywords:** chimeric antigen receptor, CAR-T cells, multiple myeloma, immunotherapy, cell therapy, tumor antigen, cancer, xenograft

## Abstract

The cell-surface protein B cell maturation antigen (BCMA, CD269) has emerged as a promising target for CAR-T cell therapy for multiple myeloma. In order to create a novel BCMA CAR, we generated a new BCMA monoclonal antibody, clone 4C8A. This antibody exhibited strong and selective binding to human BCMA. BCMA CAR-T cells containing the 4C8A scFv were readily detected with recombinant BCMA protein by flow cytometry. The cells were cytolytic for RPMI8226, H929, and MM1S multiple myeloma cells and secreted high levels of IFN-γ in vitro. BCMA-dependent cytotoxicity and IFN-γ secretion were also observed in response to CHO (Chinese Hamster Ovary)-BCMA cells but not to parental CHO cells. In a mouse subcutaneous tumor model, BCMA CAR-T cells significantly blocked RPMI8226 tumor formation. When BCMA CAR-T cells were given to mice with established RPMI8226 tumors, the tumors experienced significant shrinkage due to CAR-T cell activity and tumor cell apoptosis. The same effect was observed with 3 humanized BCMA-CAR-T cells in vivo. These data indicate that novel CAR-T cells utilizing the BCMA 4C8A scFv are effective against multiple myeloma and warrant future clinical development.

## 1. Introduction

Multiple myeloma is a cancer of plasma cells characterized by clonal proliferation in the bone marrow microenvironment [1]. Multiple myeloma is the second-most common hematologic malignancy, accounting for 5–10% of all hematologic malignancies in the USA [2,3]. Despite recent progress in treatment, multiple myeloma remains incurable with high rates of relapsed and refractory disease [3].

Investigators are now turning to immunotherapy approaches targeting B cell maturation agent (BCMA), a cell-surface protein expressed by mature B lymphocytes, plasma cells, and most cases of multiple myeloma [4]. BCMA, also known as CD269 or tumor necrosis factor receptor superfamily member 17 (TNFRSF17) [5] binds to several ligands, including BAFF (B cell activating factor) and APRIL (A proliferation inducing ligand) [6,7] to mediate cell survival through downstream NF-kappa B and MAPK/JNK signaling pathways [8,9]. BCMA has been targeted by antibodies [5] and, recently, CAR (chimeric antigen receptor)-expressing T cells [1,10,11]. CAR-T cells based on a number of monoclonal antibodies to CD19 (e.g., FMC63, SJ25C) have proven successful in the treatment of B cell malignancies [12,13,14,15,16,17]. Although BCMA CAR-T cells targeting multiple myeloma have been reported [4,18], there is a need for clinical trials with novel CAR-T cells to advance the treatment of hematological cancers [19,20].

To generate a novel BCMA-specific CAR, we generated a new BCMA-specific mAb, clone 4C8A, and characterized it in vitro. Clone 4C8A exhibited selective and high-affinity binding to BCMA, and was used to construct a single-chain variable fragment (scFv). We inserted the 4C8A scFv into a second-generation CAR, generated CAR-T cells, and measured their activity against multiple myeloma cells in vitro and in a mouse xenograft tumor model. We show that BCMA CAR-T cells based on the novel mAb 4C8A significantly decreased multiple myeloma tumor growth, demonstrating great potential for treating patients with multiple myeloma.

## 2. Results

### 2.1. BCMA mAb 4C8A Binds Selectively and with High Affinity to BCMA

**Figure 1:**
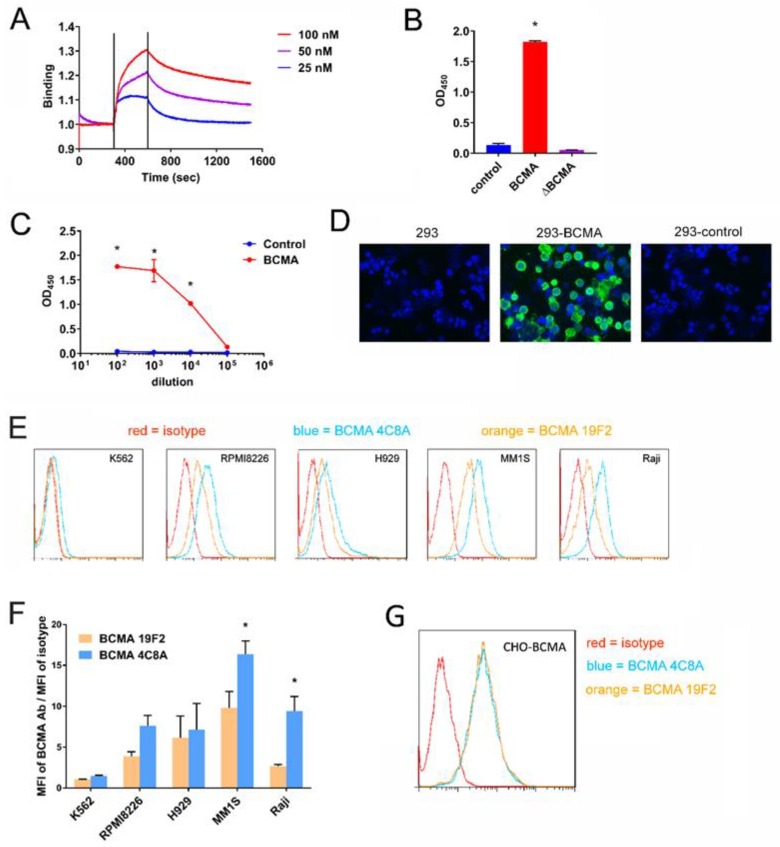
BCMA mAb 4C8A binds BCMA protein. (**A**) Binding of BCMA mAb 4C8A using Forte Bio Blitz system. BCMA mAb 4C8A was loaded onto a Blitz mouse Fc capture sensor at different concentrations. The Kd of binding was detected with Blitz software. (**B**) Binding of BCMA mAb to BCMA protein by ELISA. BCMA mAb 4C8A was incubated in ELISA plates coated with BCMA protein, BCMA protein with a C-terminal deletion of 37 residues and the first two N-terminal residues; or negative control protein, CD363 protein. * *p* < 0.0001 for BCMA protein versus BCMA and control. (**C**) Dose-dependent binding of 4C8A mAb to BCMA protein. Dilutions of BCMA mAb 4C8A were incubated in ELISA plates coated with BCMA protein or CD363 negative control protein. * *p* < 0.0001 for BCMA protein versus control. (**D**) BCMA binding to BCMA protein in 293 cells by immunofluorescent staining (IF). BCMA mAb 4C8A was incubated with HEK293 cells, HEK293 cells expressing BCMA, or HEK293 cells expressing negative control protein, CD18. Binding of BCMA mAb 4C8A was detected with Alexa Fluor 488-conjugated anti-mouse IgG. (**E**) Binding of BCMA monoclonal antibody to BCMA in multiple myeloma cells. BCMA mAb 4C8A, BCMA mAb 19F2 and a mouse IgG1 isotype control mAb were incubated with myeloma lines RPMI8226, H929, and MM1S, as well as Burkitt’s lymphoma line Raji and the BCMA-negative cell line K562. Binding of the antibodies to the cells was detected by flow cytometry with PE-conjugated anti-mouse IgG. (**F**) Quantification of binding shown in Figure 1E. To quantitate the binding in panel E, the mean fluorescence intensity (MFI) of each BCMA mAb was divided by the MFI of the isotype control mAb. * *p* < 0.05 for BCMA mAb 4C8A versus BCMA mAb 19F2 (MM1S and Raji only). (**G**) BCMA mAb 4C8A binds BCMA in CHO-BCMA cells. BCMA mAb 4C8A, BCMA mAb 19F2, and a mouse IgG1 isotype control mAb were incubated with CHO (Chinese Hamster Ovary) cells stably expressing human BCMA, and binding of the antibodies was detected by flow cytometry with PE-conjugated anti-mouse IgG.

Murine anti-human BCMA mAbs were generated by conventional methods and characterized in a variety of assays. One mAb, clone 4C8A, exhibited strong binding to BCMA, with a Kd of 2.8 nM (Figure 1A). By ELISA, clone 4C8A bound to BCMA protein but not to a control protein or to BCMA protein containing a deletion of the C-terminal 37 residues (Figure 1B). BCMA binding was dose-dependent (Figure 1C). By immunofluorescence, clone 4C8A bound to HEK293 cells expressing BCMA but not to parental HEK293 cells or to HEK293 cells transiently expressing negative control protein (Figure 1D). Thus, BCMA 4C8A antibody detects BCMA protein with high affinity and specificity.

### 2.2. BCMA Monoclonal 4C8A Antibody Specifically Recognizes BCMA in Multiple Myeloma

To detect BCMA monoclonal antibody binding to BCMA in multiple myeloma cells, we performed FACS analysis on several multiple myeloma cell lines: RPMI8226, H929, and MM1S with BCMA antibody 4C8A and also on negative control BCMA-negative K562 cell lines. By flow cytometry, clone 4C8A bound to multiple myeloma lines, as well as Burkitt’s B-lymphoma Raji cells, but not to BCMA-negative K562 control cells (Figure 1E). Binding was generally greater for clone 4C8A than a commercially-available BCMA mAb, clone 19F2 (Figure 1F). Both mAbs exhibited similar binding to CHO cells expressing human BCMA protein (Figure 1G) demonstrating high specificity of both antibodies to BCMA.

**Figure 2:**
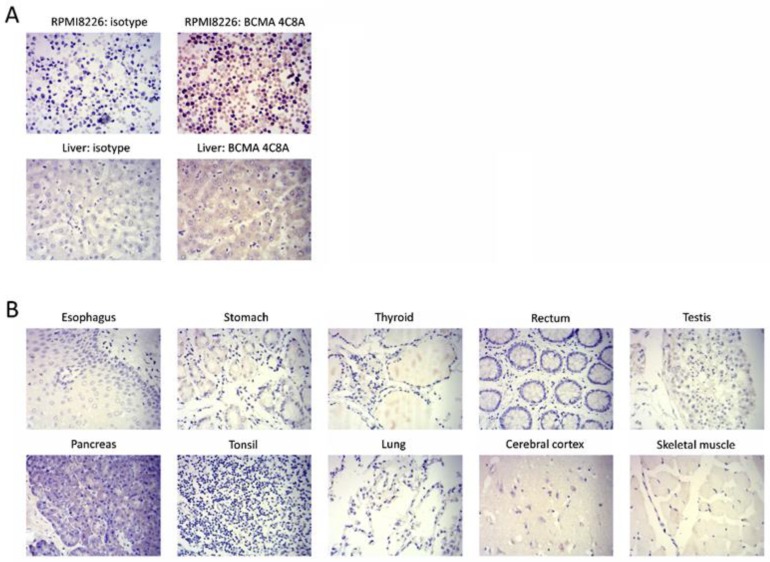
Immunohistochemical staining of normal human tissues by BCMA 4C8A mAb. (**A**) BCMA 4C8A but not the isotype control mAb stained (brown color) RPMI8226 myeloma cells and normal human liver. (**B**) BCMA 4C8A did not stain any other normal human tissues. Blue color: nucleus counterstain. Original magnification 400×.

To detect specificity of BCMA in human tissues, the IHC (Immunohistochemistry staining) was performed on several normal tissues. By IHC, clone 4C8A bound to RPMI8226 cells and normal human liver, but not to any other normal human tissues (Figure 2), confirming the specificity of BCMA expression. In addition, we detected positive BCMA staining in primary bone marrow myeloma tissue sample but not in negative control adrenal gland tissue sample (Figure S1) that additionally supports high specificity of BCMA monoclonal antibody to multiple myeloma cells.

### 2.3. CAR-T Cells Generated with BCMA 4C8A Antibody ScFv Recognize BCMA Protein

**Figure 3:**
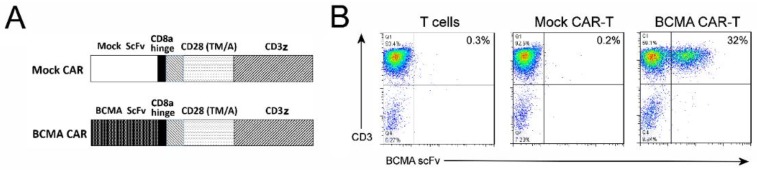

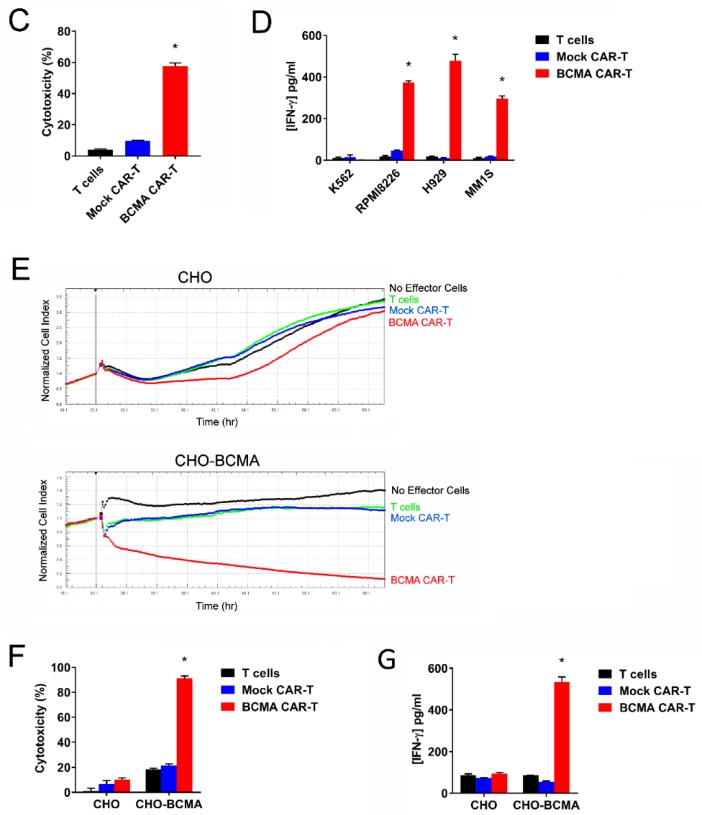
Characterization of BCMA 4C8A CAR-T cells in vitro. (**A**) Diagram of the BCMA and mock CARs, with the following regions (from N-terminus to C-terminus): scFv, CD8 hinge, CD28 transmembrane domain, CD28 costimulatory domain, CD3 zeta activation domain. (**B**) FACS analysis with BCMA protein detects BCMA-CAR expression. BCMA CAR-T cells, mock CAR-T cells, and non-transduced T cells were analyzed by flow cytometry, using an APC-conjugated anti-CD3 mAb (*Y*-axis) and biotinylated BCMA protein (*X*-axis). The percentage of cells binding to both the CD3 mAb and the BCMA protein (i.e., BCMA CAR-T cells) is shown in the upper right quadrant. (**C**) BCMA-CAR-T cells lyse RPMI8226 multiple myeloma cells by LDH assay. BCMA CAR-T cells, mock CAR-T cells, and non-transduced T cells were incubated with RPMI8226 myeloma cells, and the levels of LDH released into the medium was measured by a colorimetric enzyme assay as described in Materials and Methods. The LDH assay demonstrates specific LDH release by target cells. * *p* < 0.0001 for BCMA CAR-T cells versus mock CAR-T cells and non-transduced T cells. (**D**) BCMA-CAR-T cells secrete IFN-γ against multiple myeloma cells. BCMA CAR-T cells, mock CAR-T cells and non-transduced T cells were incubated with myeloma lines: RPMI8226, H929, and MM1S, as well as the BCMA-negative cell line K562. The levels of IFN-γ released into the medium was measured by ELISA; * *p* < 0.0001 for BCMA CAR-T cells versus mock CAR-T cells and non-transduced T cells. (**E**) BCMA-CAR-T cells killed CHO-BCMA cells by real-time cytotoxicity assay (RTCA). BCMA CAR-T cells, mock CAR-T cells, and non-transduced T cells were added to monolayers of CHO cells (top row) and CHO-BCMA cells (bottom row), and the impedance proportional to cell number of the monolayers was monitored over time. The average of three replicates is shown. (**F**) Quantitation of cytotoxicity at the end of the RTCA assay in panel E; * *p* < 0.0001 for BCMA CAR-T cells versus mock CAR-T cells and non-transduced T cells. (**G**) BCMA-CAR-T cells significantly increased IFN-gamma secretion against CHO-BCMA cells. The levels of IFN-gamma released into the RTCA medium was measured by ELISA; * *p* < 0.0001 for BCMA CAR-T cells versus mock CAR-T cells and non-transduced T cells.

The sequences of clone 4C8A’s heavy and light chain variable regions were determined and used to construct a single-chain variable fragment (scFv). The scFv was inserted into a chimeric antigen receptor (CAR) cassette next to a CD8 hinge region, transmembrane and costimulatory domains from human CD28, and the activation domain from human CD3 zeta (Figure 3A). For a negative control, a ‘mock’ scFv from a mAb specific for control intracellular protein was likewise inserted into the CAR cassette. The CARs were inserted into a lentiviral expression vector downstream of the human EF1a promoter and CD8 signal sequence. After one week of expansion in culture, the transduced T cells with CAR lentivirus were analyzed by flow cytometry, using biotinylated BCMA protein.

The BCMA protein bound to 30–40% of BCMA CAR-T cells but did not bind to mock CAR-T cells or control non-transduced T cells (Figure 3B). This demonstrates that BCMA-CAR-T cells expressed BCMA scFv and recognized BCMA.

### 2.4. CAR-T Cells Based on BCMA Clone 4C8A Effectively Kill BCMA^+^ Cancer Cells and Secreted IFN-Gamma

The CAR-T cells were tested functionally in several ways. First, the cells were co-cultured with RPMI8226 target cells and RPMI8226 lysis was measured by lactate dehydrogenase (LDH) release. BCMA CAR-T cells, but not mock CAR-T cells or non-transduced T cells, effectively lysed the RPMI8226 cells (Figure 3C). High killing activity of BCMA-CAR-T cells was also observed with other target multiple myeloma cell lines: MM1S and H929. The CAR-T cells were also evaluated for IFN-γ production after co-culture with multiple myeloma cell lines; BCMA CAR-T cells produced IFN-γ in response to RPMI8226, H929, and MM1S cells, but not in response to BCMA-negative K562 control cells (Figure 3D).

To test BCMA-dependent activity of CAR-T cells, the CAR-T cells were assessed for cytotoxicity with CHO-BCMA target cells by real-time cellular analysis (RTCA). BCMA CAR-T cells, but not mock CAR-T cells or non-transduced cells, substantially decreased the impedance of the CHO-BCMA monolayer, indicative of cytolysis (Figure 3E,F). BCMA CAR-T cells did not kill parental CHO cells, indicating that the cytotoxicity for CHO-BCMA cells was BCMA-dependent (Figure 3E,F). Analysis of the medium from the RTCA assay indicated that BCMA CAR-T cells produced IFN-γ in response to CHO-BCMA but not CHO cells (Figure 3G). These data show BCMA-dependent cytotoxicity of BCMA-CAR-T cells and secretion of IFN-γ.

### 2.5. BCMA Clone 4C8A CAR-T Cells Block Subcutaneous RPMI8226 Xenograft Tumor Growth In Vivo

**Figure 4:**
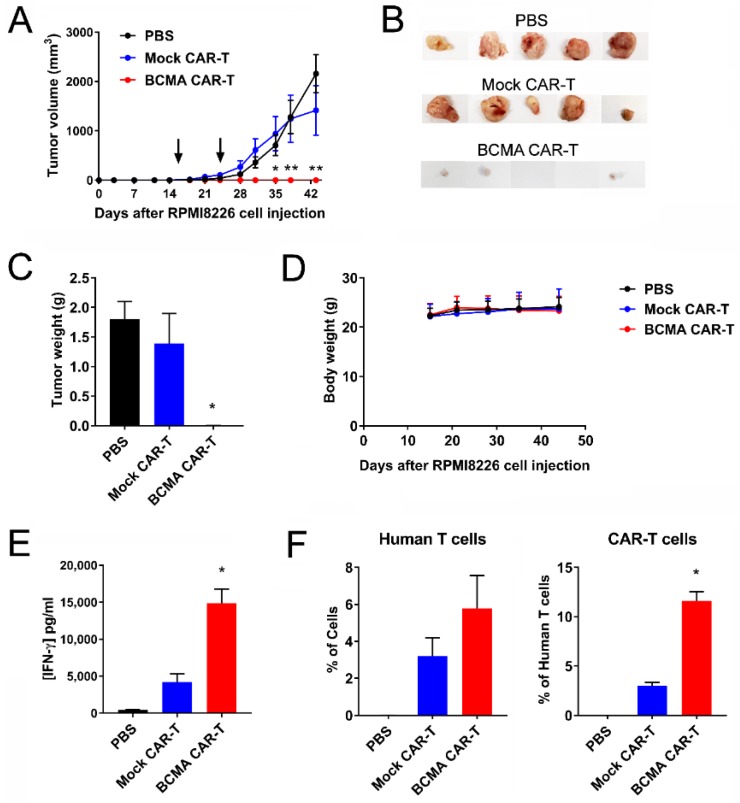
BCMA 4C8A CAR-T cells block RPMI 8226 xenograft tumor growth. (**A**) NSG (NOD Scid gamma) mice were injected subcutaneously with RPMI8226 myeloma cells and tumor size was measured bi-weekly with calipers. On days 16 and 24 (arrows), the mice received BCMA CAR-T cells, mock CAR-T cells, or PBS intravenously; * *p* < 0.01, ** *p* < 0.0001 for BCMA CAR-T cells versus mock CAR-T cells and PBS. (**B**) The tumors were excised and photographed. (**C**) The weight of the tumors significantly decreased in BCMA-treated mice. * *p* < 0.05 for BCMA CAR-T cells versus mock CAR-T cells and PBS. (**D**) BCMA-CAR-T cells did not change mice weight. (**E**) BCMA-treated mice had significantly increased level of IFN-gamma in the blood plasma. Human IFN-γ levels were measured in the plasma by ELISA at the end of the study; * *p* < 0.0001 for BCMA CAR-T cells versus mock CAR-T cells and PBS. (**F**) The level of human T cells and CAR-T cells in the blood of BCMA-treated mice was significantly increased versus control treated mice. The peripheral blood cells were analyzed by flow cytometry at the end of the study for binding to human BCMA protein and an antibody specific for human CD3. The percentage of cells binding to the CD3 mAb is shown on the left, and the percentage of those human T cells that also bound to the BCMA protein is shown on the right; * *p* < 0.0001 for BCMA CAR-T cells versus mock CAR-T cells.

BCMA 4C8A CAR-T cells were tested in an NSG (NOD Scid gamma) mouse subcutaneous tumor model using RPMI8226 cells. CAR-T cells were administered intravenously at 16 and 24 days after RPMI8226 injection, and tumor size was measured with calipers. BCMA CAR-T cells, but not mock CAR-T cells or PBS, blocked the RPMI8226 xenograft tumor growth (Figure 4A). The images of tumors are shown on (Figure 4B). The tumor weight of BCMA-treated mice was significantly decreased compared to control mock-treated mice (Figure 4C). BCMA CAR-T cells did not affect mouse weight (Figure 4D). Levels of human IFN-γ in the bloodstream at the end of the study were significantly higher for BCMA CAR-T cell-treated mice than for the control mice, indicative of greater IFN-γ production by the BCMA CAR-T cells (Figure 4E).

Human T cells were detected in the bloodstream of both BCMA CAR-T cell-treated mice and mock CAR-T cell-treated mice (Figure 4F). Importantly, there was a significantly increased level of BCMA protein staining in BCMA-treated mice blood supporting increased level of BCMA-CAR-T cells in mouse blood (Figure 4F). This demonstrates significant inhibition of RPMI8226 xenograft tumor formation and growth by BCMA-CAR-T cell in vivo.

### 2.6. BCMA Clone 4C8A CAR-T Cells Block Growth of Subcutaneous Established RPMI8226 Xenograft Tumors In Vivo

**Figure 5:**
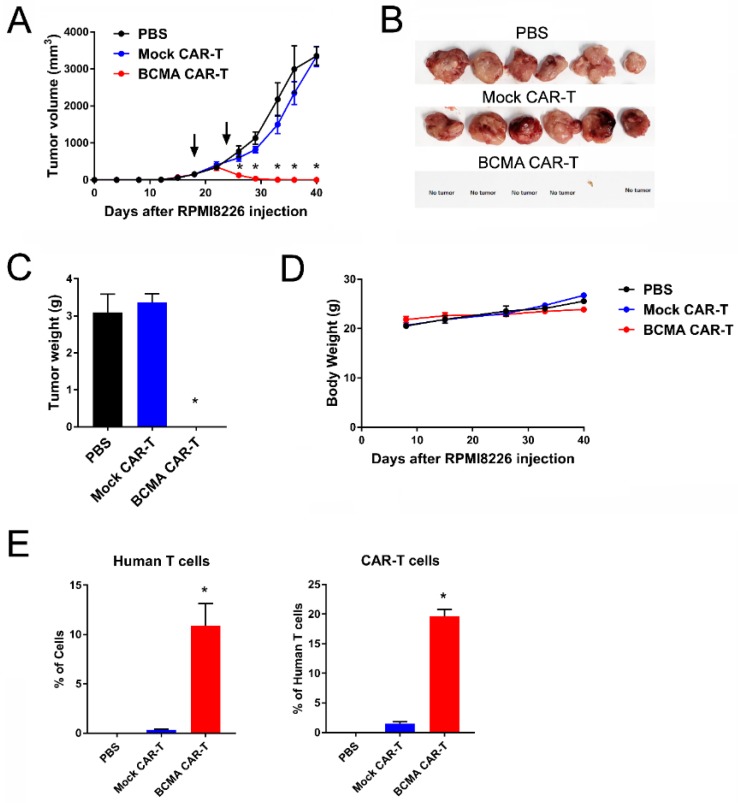
BCMA-CAR-T cells significantly block growth of subcutaneous established (150 mm^3^) RPMI8226 xenograft tumors. (**A**) NSG mice were injected subcutaneously with RPMI8226 myeloma cells and tumor size was measured bi-weekly with calipers. On days 18 and 24 (arrows), the mice received PBS, BCMA CAR-T cells or mock CAR-T cells intravenously; * *p* < 0.0001 for BCMA CAR-T cells versus PBS and mock CAR-T cells. (**B**) The tumors were excised and photographed. (**C**) The excised tumors were weighed; * *p* < 0.0001 for BCMA CAR-T cells versus mock CAR-T cells. (**D**) The mice were weighed weekly during the study. (**E**) The peripheral blood cells were analyzed by flow cytometry at the end of the study for binding to human BCMA protein and an antibody specific for human CD3. The percentage of cells binding to the CD3ζmAb is shown on the left, and the percentage of those human T cells that also bound to the BCMA protein is shown on the right; * *p* < 0.05 for Human T cells (BCMA versus mock and PBS), *p* < 0.0001 for CAR-T cells (BCMA versus mock).

To further explore anti-tumor activities of BCMA-4C8A-CAR-T cells, NSG mice with established subcutaneous RPMI8226 tumors were treated with BCMA-CAR-T cells. When the tumors were approximately 150 mm^3^, CAR-T cells were injected intravenously at days 18 and 24. BCMA CAR-T cells significantly decreased tumor size in mice (Figure 5A); at the end of the study, only one tiny tumor was found among the mice (Figure 5B,C). In the mice treated with PBS or the mock CAR-T cells, tumors continued to enlarge over time (Figure 5A–C). BCMA CAR-T cells did not affect mouse weight (Figure 5D). Significantly more human T cells were detected in the bloodstream of BCMA CAR-T cell-treated mice than in mock CAR-T cell-treated mice, and nearly 20% of these human T cells were CAR-T cells (Figure 5E). Thus, BCMA-CAR-T cells significantly blocked established RPMI8226 xenograft tumor growth consistent with significantly increased number of BCMA-CAR-T cells in the mice blood.

**Figure 6:**
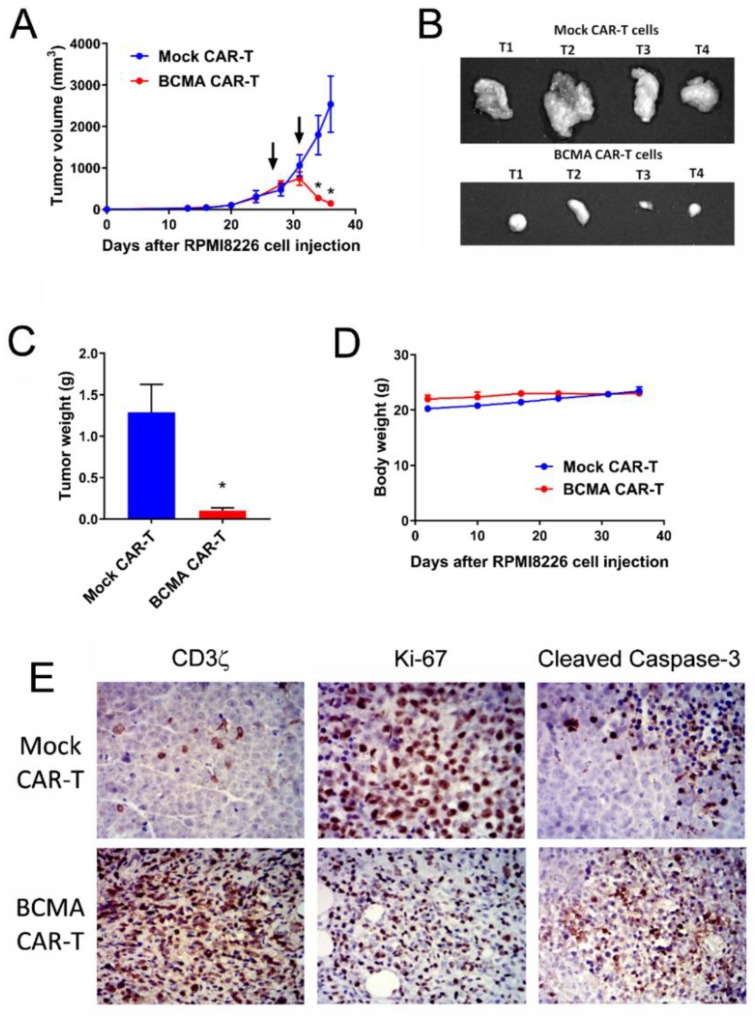
BCMA-CAR-T cells significantly block growth of subcutaneous large size established (500 mm^3^). RPMI8226 xenograft tumors. (**A**) NSG mice were injected subcutaneously with RPMI8226 myeloma cells and tumor size was measured bi-weekly with calipers. On days 27 and 31 (arrows), the mice received BCMA CAR-T cells or mock CAR-T cells intravenously; * *p* < 0.0001 for BCMA CAR-T cells versus mock CAR-T cells. (**B**) The tumors were excised and photographed. (**C**) The excised tumors were weighed; * *p* < 0.05 for BCMA CAR-T cells versus mock CAR-T cells. (**D**) The mice were weighed weekly during the study. (**E**) Sections of the tumors were stained immunohistochemically (brown color) with antibodies specific for human CD3ζ, Ki-67 and cleaved caspase-3. Top row: tumors from mice receiving mock CAR-T cells. Bottom: tumors from mice receiving BCMA CAR-T cells. Blue color: nucleus counterstain. Original magnification 400×.

To detect the effect of BCMA-CAR-T cell on the growth of large established tumors, BCMA 4C8A CAR-T cells were administered on days 27 and 31, when the tumors reached 500 mm^3^. Mock CAR-T cells failed to halt tumor growth, but the BCMA CAR-T cells caused a rapid and sustained decrease in tumor growth (Figure 6A–C). Moreover, BCMA CAR-T cells did not affect mouse weight (Figure 6D).

Tumors were analyzed by IHC at the end of the study. Tumors from mice treated with mock CAR-T cells consisted primarily of dividing tumor cells with high Ki67 staining, with occasional human T cells (human CD3 staining) and rare small foci of apoptotic tumor cells (cleaved caspase-3 stain) (Figure 6E, top row). In contrast, tumors from mice treated with BCMA CAR-T cells had decreased Ki67 tumor staining associated with vast numbers of dividing human T cells (Ki-67 and CD3 stains) and large regions filled with apoptotic tumor cells, reflecting CAR-T cell expansion and tumor cell killing (Figure 6E, bottom row). BCMA expression decreased in BCMA-treated tumors. Thus, BCMA-CAR-T cells significantly blocked growth of large established RPMI 8226 xenograft tumors.

### 2.7. Humanized BCMA Clone 4C8A CAR-T Cells Block Multiple Myeloma Xenograft Tumor Growth In Vivo

**Figure 7:**
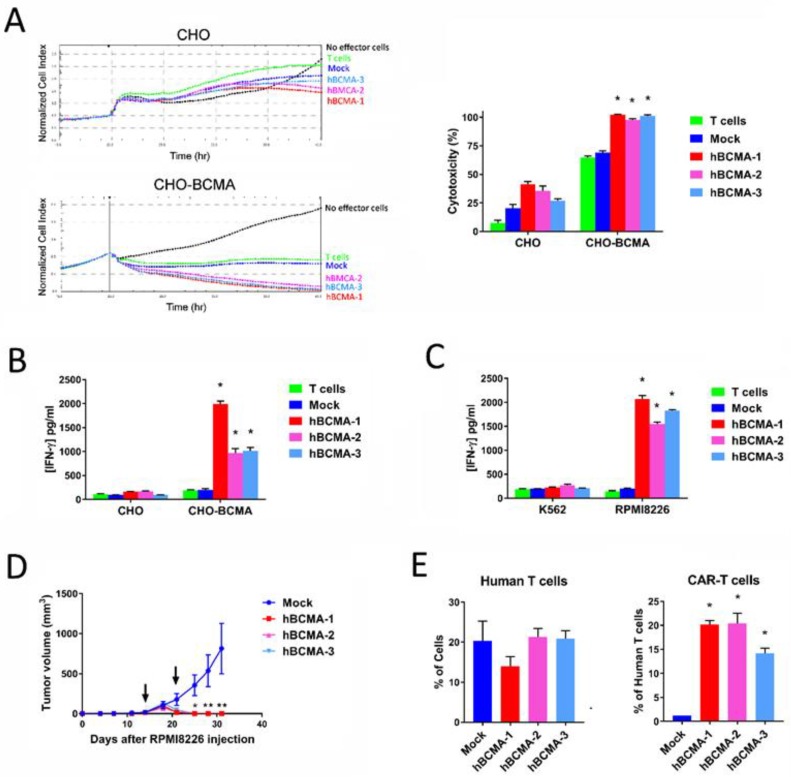
Characterization of humanized BCMA 4C8A CAR-T cells. (**A**) Three humanized clones were evaluated for cytolytic activity by RTCA on CHO and CHO-BCMA target cells at an E:T ratio of 10:1. The quantitation of cytotoxicity at the end of the assay is shown on the right; * *p* < 0.0001 for all 3 clones versus mock and non-transduced T cells. (**B**) The levels of IFN-γ produced in the RTCA assay were quantitated by ELISA; * *p* < 0.0001 for all 3 clones versus mock and non-transduced T cells. (**C**) The humanized BCMA 4C8A CAR-T cells were cultured overnight with BCMA-positive RPMI8226 myeloma cells and BCMA-negative K562 cells, then the levels of IFN-γ produced in the culture were quantitated by ELISA; * *p* < 0.0001 for all 3 clones versus mock and non-transduced T cells. (**D**) The humanized BCMA 4C8A CAR-T cells were tested in the RPMI8226 tumor model for inhibition of tumor growth; * *p* = 0.043 and ** *p* ≤ 0.005 for all 3 clones versus mock. (**E**) The peripheral blood cells were analyzed by flow cytometry at the end of the study for binding to human BCMA protein and antibodies specific for human T (CD4+/CD8+) cells. The percentage of cells binding to the CD4 mAb is shown on the left, and the percentage of those human T cells that also bound to the BCMA protein is shown on the right; * *p* < 0.05 for Human T and BCMA-CAR-T cells (BCMA versus Mock).

We humanized the 4C8A scFv and tested 3 different clones for activity in the assays described above. All three humanized BCMA-CAR-T cells (BCMA-h1, h2, h3) were cytolytic for CHO-BCMA cells but not CHO cells (Figure 7A), and produced IFN-γ in response to CHO-BCMA but not CHO cells (Figure 7B). In addition, all 3 clones produced IFN-γ in response to the BCMA-positive myeloma cell line RPMI8226 but not in response to the BCMA-negative cell line K562 (Figure 7C). In the RPMI8226 xenograft tumor model, all 3 clones significantly blocked tumor growth (Figure 7D) and did not affect mouse weight. Human T cells were detected in the bloodstream of both mock-CAR-T cell and humanized BCMA CAR-T cell-treated mice (Figure 7E). Importantly, there was a significantly increased level of BCMA protein staining in BCMA-treated mice blood (Figure 7E). These data clearly demonstrate that humanized BCMA-CAR-T cells killed multiple myeloma cells in vitro and significantly inhibited multiple myeloma xenograft tumor growth in vivo.

## 3. Discussion

Multiple myeloma is an aggressive hematological cancer warranting novel immunotherapy approaches such as targeting BCMA which is highly expressed in this malignancy. In this report, we show that BCMA-specific CAR-T cells using the novel 4C8A scFv have great therapeutic potential for multiple myeloma.

We demonstrate that BCMA clone 4C8A binds with high affinity to the whole BCMA extracellular domain (amino acids 1–54 a.a), but not to a fragment of BCMA consisting of residues 3–17 a.a. We initially generated monoclonal antibodies against this fragment, but the antibodies did not exhibit sufficient specificity for multiple myeloma cells. This suggests that using the whole extracellular domain as an immunogen is a better approach for generating BCMA monoclonal antibodies. Clone 4C8A stained multiple myeloma cells but did not stain the vast majority of normal cells or other types of cancers, demonstrating the specificity of the antibody to multiple myeloma. Weak staining was observed in liver, where BCMA expression has previously been noted [21]. APRIL-BCMA interactions in normal liver and hepatocellular carcinoma trigger JNK2 and FOXO3A signaling that regulate hepatocyte proliferation [21]. Thus, the BCMA 4C8A antibody can be used for therapeutic applications in multiple myeloma and potentially in hepatocellular carcinoma.

To use the 4C8A antibody for therapeutics, we generated BCMA 4C8A CAR-T cells and observed that human BCMA protein could be used to stain CAR-positive cells. BCMA 4C8A CAR-T cells killed BCMA-positive multiple myeloma cell lines RPMI8226, MM1S, and H929 but did not kill BCMA-negative K562 leukemia control cells. In addition, BCMA 4C8A CAR-T cells killed CHO-BCMA cells but did not kill parental CHO cells. Moreover, BCMA 4C8A CAR-T cells secreted significantly higher levels of IFN-gamma in response to BCMA-positive cells than to BCMA-negative cells. These data demonstrate the potent and specific in vitro activity of BCMA-CAR-T cells.

We also show that BCMA 4C8A CAR-T cells significantly decreased NSG mouse RPMI8226 xenograft tumor growth, whereas mock CAR-T cells had no effect. When BCMA 4C8A CAR-T cells were applied early, RPMI8226 xenograft tumor was completely blocked. When BCMA 4C8A CAR-T cells were applied at later time points to mice containing either mid-sized or large tumors (150–500 mm^3^), tumor size significantly decreased over time. IHC staining of the tumors indicated massive infiltration by dividing human T cells, activation of pro-apoptotic caspase 3 and a severe decrease in dividing tumor cells. At the end of the studies, BCMA 4C8A CAR-T cells were detected in the blood of the treated mice, demonstrating the in vivo persistence of the CAR-T cells. These data clearly demonstrate a potent anti-tumor efficacy of BCMA 4C8A CAR-T cells in vivo.

To test humanized BCMA CAR-T cells, we humanized the BCMA 4C8A scFv as described in [22] and generated three humanized CAR-T cells. The 3 humanized CAR-T cells killed CHO-BCMA cells but did not affect CHO cells. Humanized BCMA-CAR-T cells secreted IFN-γ in response to CHO-BCMA but not CHO cells (Figure 7). In addition, humanized BCMA 4C8A CAR-T cell demonstrated high efficacy in vivo. These data support high efficacy of BCMA 4C8A and humanized BCMA 4C8A-CAR-T cells against multiple myeloma.

Multiple myeloma cells express other cell-surface tumor antigens that can be targeted, such as CS1, CD38, and CD33 [10]. To increase CAR-T efficacy and specificity, novel bispecific CAR-T cells can be used to target multiple myeloma [23]. This approach was used recently for CD19 and CD22 in B cell leukemia; CD19-CD22 bispecific CAR-T cells were more effective than either CAR-T cell alone [24]. The approach was also demonstrated for CD19-CD20 tandem and bispecific CAR-T cells [25,26]. Thus, novel monospecific and bispecific CAR-T cells can be used in future clinical studies. Novel switches and split designs such as Syn-Notch CAR-T cells [27] or SUPRA-CAR-T cells [28] can also be used for multiple myeloma. In addition, combination of CAR-T cells with checkpoint inhibitors and microenvironment targeting agents can be applied for more effective therapy in the clinic [29,30].

## 4. Materials and Methods

### 4.1. Cells, Primary Tissues

Cell lines RPMI8226, H929, MM1S, Raji, K562, and CHO were purchased from the ATCC (Manassas, VA, USA) and cultured either in DMEM (GE Healthcare, Chicago, IL, USA) or in RPMI-1640 medium (Thermo Fisher, Waltham, MA, USA) containing 10% FBS (AmCell, Mountain View, CA, USA). CHO-BCMA cells were purchased from BPS Bioscience (San Diego, CA, USA) and cultured in Ham’s F12K medium containing 10% FBS and 1 mg/mL Geneticin (Thermo Fisher). Human peripheral blood mononuclear cells (PBMC) were isolated from whole blood obtained in the Stanford Hospital Blood Center, Stanford according to IRB-approved protocol. PBMC were isolated by density sedimentation over Ficoll-Paque (GE Healthcare). Bone marrow primary myeloma tissue and adrenal gland control tissue samples were obtained from BioMAX Inc (Derwood, MD, USA).

### 4.2. Generation of A BCMA-Specific Monoclonal Antibody

BALB/c mice of 6–8 week old were immunized by subcutaneous injection with a peptide containing the extracellular BCMA domain (residues 1–54) of human BCMA linked to a tag. To generate hybridoma splenocytes from immunized mice were fused with SP/0 myeloma cells using PEG and then hypoxantine (HAT) medium selection. Hybridomas were diluted to obtain single clones on 96-well plates and were screened by ELISA for positive clones using the immunogen versus an unrelated tagged peptide. Several positive hybridoma clones were further cultured and expanded to produce anti-BCMA antibodies. The supernatant of these antibodies were collected and purified through Protein G affinity capture then size exclusion chromatography.

### 4.3. Generation of CAR-Encoding Lentivirus

A DNA encoding the BCMA CAR was synthesized and subcloned into a third-generation lentiviral vector, Lenti CMV-MCS-EF1a-puro by Syno Biological (Beijing, China). Three different humanized BCMA ScFv (BCMA-h1, h2 and h3) were generated as described in [22] and used for CAR lentivirus. Ten million growth-arrested HEK293FT cells (Thermo Fisher) were seeded into T75 flasks and cultured overnight, then transfected with the pPACKH1 Lentivector Packaging Mix (System Biosciences, Palo Alto, CA, USA) and 10 µg of the lentiviral vector using the CalPhos Transfection Kit (Takara, Mountain View, CA, USA). The next day the medium was replaced with fresh medium, and 48 h later the lentivirus-containing medium was collected. The medium was cleared of cell debris by centrifugation at 2100× *g* for 30 min. The virus particles were collected by centrifugation at 112,000× *g* for 100 min, suspended in AIM V medium, aliquoted and frozen at −80 °C. The titers of the virus preparations were determined by quantitative RT-PCR using the Lenti-X qRT-PCR kit (Takara) according to the manufacturer’s protocol and the 7900HT thermal cycler (Thermo Fisher). The lentiviral titers were >1 × 10^8^ pfu/mL.

### 4.4. Generation and Expansion of CAR-T Cells

PBMC were suspended at 1 × 10^6^ cells/mL in AIM V-AlbuMAX medium (Thermo Fisher) containing 10% FBS and 10 ng/mL IL-2 (Thermo Fisher), mixed with an equal number (1:1 ratio) of CD3/CD28 Dynabeads (Thermo Fisher), and cultured in non-treated 24-well plates (0.5 mL per well). At 24 and 48 h, lentivirus was added to the cultures at a multiplicity of infection (MOI) of 5, along with 1 µL of TransPlus transduction enhancer (AlStem, San Francisco, CA, USA). As the T cells proliferated over the next 10–12 days, the cells were counted every 2–3 days and fresh medium with 10 ng/mL IL-2 was added to the cultures to maintain the cell density at 1 to 3 × 10^6^ cells/mL.

### 4.5. Flow Cytometry

To measure CAR expression, 0.25 million cells were suspended in 100 µL of buffer (PBS containing 2 mM EDTA pH 8 and 0.5% BSA) and incubated on ice with 1 µL of human serum (Jackson Immunoresearch, West Grove, PA, USA) for 10 min. Then 0.3 µg of biotinylated human BCMA protein (Acro Biosystems, Newark, DE, USA) was added, and the cells were incubated on ice for 30 min. The cells were rinsed with 3 mL of buffer and suspended in 100 µL of buffer. Then 1 µL of phycoerythrin (PE)-conjugated streptavidin (BD Biosciences, San Jose, CA, USA), 1 µL of allophycocyanin (APC)-labeled anti-CD3 (BioLegend, San Diego, CA, USA) and 2 µL of 7-aminoactinomycin D (7-AAD) solution (BioLegend) were added, and the cells were incubated on ice for 30 min. The cells were rinsed with 3 mL of buffer, then suspended in buffer and acquired on a FACSCalibur (BD Biosciences). Cells were analyzed first for light scatter versus 7-AAD staining, then the 7-AAD^−^ live gated cells were plotted for anti-CD3 staining versus BCMA protein staining. For the mouse tumor studies, 100 µL of blood obtained via retro-orbital puncture was depleted of erythrocytes by incubation with 3 mL of RBC lysing solution (150 mM NH_4_Cl, 10 mM NaHCO_3_, 1 mM EDTA pH 8) for 5 min. The leukocytes were collected by centrifugation, rinsed with 3 mL of cold buffer, suspended in 100 µL of buffer, incubated on ice with 1 µL of mouse serum (Jackson Immunoresearch) for 10 min, and stained with BCMA protein and anti-CD3 as indicated above.

### 4.6. Lactate Dehydrogenase (LDH) Assay

LDH assay was performed using Promega Cytotox 96 Non-radioactive assay kit according to the manufacturer’s protocol. Cytotoxicity is calculated using the following formula Experimental-Target Spontaneous-Effector Spontaneous/Target Maximum-Target Spontaneous LDH Release × 100%.

### 4.7. Blitz Binding Assay

The Forte Bio binding assay was performed using Blitz machine that measures bio layer interferometry, BLI according to the manufacturer’s protocol. Kd was detected using BCMA antibody, protein-specific sensors, and human BCMA protein with Blitz system and software.

### 4.8. Cytokine Induction Assay

Target cells (RPMI8226, H929, MM1S, K562) were cultured with the effector cells (CAR-T cells or non-transduced T cells) at a 1:1 ratio (1 × 10^4^ cells each) in U-bottom 96-well plates with 200 µL of AIM V-AlbuMAX medium containing 10% FBS, in triplicate. After 16 h, the top 150 µL of medium was transferred to V-bottom 96-well plates and centrifuged at 300× *g* for 5 min to pellet any residual cells. The top 120 µL of supernatant was transferred to a new 96-well plate and analyzed by ELISA for human IFN-γ levels using a kit from R&D Systems (Minneapolis, MN, USA) according to the manufacturer’s protocol

### 4.9. Real-Time Cytotoxicity Assay (RTCA)

Adherent target cells (CHO or CHO-BCMA) were seeded into 96-well E-plates (Acea Biosciences, San Diego, CA, USA) at 1 × 10^4^ cells per well and monitored in culture overnight with the impedance-based real-time cell analysis (RTCA) xCELLigence system (Acea Biosciences). The next day, the medium was removed and replaced with AIM V-AlbuMAX medium containing 10% FBS ± 1 × 10^5^ effector cells (CAR-T cells or non-transduced T cells), in triplicate. The cells in the E-plates were monitored for another one to two days with the RTCA system, and impedance was plotted over time. Cytolysis was calculated as (impedance of target cells without effector cells−impedance of target cells with effector cells) × 100/impedance of target cells without effector cells.

### 4.10. Mouse RPMI8226 Xenograft Tumors

Six-week old male NSG mice (*Jackson Laboratories*, Bar Harbor, ME) were housed and manipulated in strict accordance with the Institutional Animal Care and Use Committee (IACUC). Each mouse was injected subcutaneously on day 0 with 100 µL of 1 × 10^7^ RPMI8226 cells/mice. CAR-T cells were injected intravenously twice (1 × 10^7^ cells/mice each day) on days 16 or 18 and 24 (with small xenograft tumors) or days 27 and 31 with large (500 mm^3^) tumors (1 × 10^7^ cells/mice each injection). Tumor sizes were measured with calipers twice-weekly and tumor volume (in mm^3^) was determined using the formula W^2^L/2, where W is tumor width and L is tumor length. At the end of the studies, blood was collected by cardiac puncture and stained by flow cytometry as indicated above. In addition, plasma was isolated from the blood by centrifugation and analyzed by ELISA for human IFN-γ levels (R&D Systems). Tumors were excised and fixed in 4% paraformaldehyde, then embedded in paraffin wax and stained by immunohistochemistry.

For the mouse tumor study with humanized BCMA-CAR-T cells, RPMI8226 cells were injected subcutaneously using NSG mice, then humanized BCMA-CAR-T cells and mock control cells were injected by i.v. on days 14 and 21. At the end of the study, peripheral blood leukocytes were prepared and blocked as described above, then stained with 0.3 µg of BCMA protein coupled to an Fc fragment of human IgG (Acro Biosystems). After rinsing with 3 mL of buffer, the cells were stained with 1 µL of PE goat anti-human IgG (Jackson Immunoresearch), 2 µL of 7-AAD solution, and a mixture of either 3 µL FITC anti-CD4 and 2 µL APC anti-CD8 (Biolegend). The cells were rinsed with 3 mL of buffer, then suspended in buffer and acquired on the cytometer.

### 4.11. Immunohistochemistry (IHC)

Tumor tissue sections (4 µM) were deparaffinized in xylenes twice for 10 min, then hydrated in graded alcohols and rinsed in PBS. Antigen retrieval was performed for 20 min in a pressure cooker using 10 mM citrate buffer, pH 6.0. The sections were cooled, rinsed with PBS, incubated in a 3% H_2_O_2_ solution for 10 min, and rinsed with PBS. The tissue sections were incubated in goat serum for 20 min and then incubated with primary antibodies: BCMA clone 4C8A, mouse IgG2a isotype control (BioLegend, San Diego, CA, USA), CD3ζ clone 4B10 (Promab Biotechnologies, Richmond CA, USA), Ki-67 clone 4A1 (Promab Biotechnologies), rabbit anti-cleaved caspase-3 clone Asp175 (Cell Signaling Technology, Danvers, MA, USA), or rabbit IgG (Jackson Immunoresearch) overnight at 4 °C. The sections were rinsed with PBS, incubated with biotin-conjugated goat anti-mouse IgG or anti-rabbit IgG for 10 min, rinsed with PBS, incubated with streptavidin-conjugated peroxidase for 10 min, and rinsed with PBS. Finally, the sections were incubated in DAB substrate solution for 2–5 min, immersed in tap water, counterstained with hematoxylin, rinsed with water, and dehydrated in graded alcohols and xylenes. Coverslips were mounted with glycerin. All reagents except those noted were from MaiXin.BIO (Fuzhou, China). Images were acquired on a Motic DMB5-2231PL microscope with Images Plus 2.0 software.

### 4.12. Statistical Analysis

Data were analyzed and plotted with Prism software (GraphPad, San Diego, CA, USA). Comparisons between two groups were performed by unpaired Student’s *t*-test. Comparisons between three or more groups were performed by one-way ANOVA or two-way ANOVA with Tukey’s or Sidak’s post hoc test. The difference with *p* < 0.05 was considered significant.

## 5. Conclusions

In conclusion, we show that novel BCMA 4C8A CAR-T cells humanized BCMA 4C8A CAR-T cells exhibit potent and specific activity in vitro and in vivo. It will be important to test these CAR-T cells in future clinical trials.

## Supplementary Materials

The following are available online at http://www.mdpi.com/2072-6694/10/9/323/s1, Figure S1: The IHC staining with BCMA Ab 4C8A and isotype control (1:300 dilution) was performed on primary bone marrow myeloma tissue sample and negative control adrenal gland pheochromocytoma tissue.
