# Supplementary material for: CAR-T Cells Based on Novel BCMA Monoclonal Antibody Block Multiple Myeloma Cell Growth
Source: Cancers (Basel). 2018 Sep 11;10(9):323. doi: 10.3390/cancers10090323 (PMC6162381; doi:10.3390/cancers10090323)
Supplement: Supplementary file 1 [file cancers-10-00323-s001.pdf]

cancers

Supplementary Materials: CAR-T Cells Based on Novel BCMA Monoclonal Antibody Block Multiple Myeloma Cell Growth

Robert Berahovich [1], Hua Zhou [1], Shirley Xu [1], Yuehua Wei [1], Jasper Guan [1], Jian Guan [1], Hizkia Harto [1], Shuxiang Fu [2], Kaihuai Yang [2], Shuying Zhu [2], Le Li [1,2], Lijun Wu [1] and Vita Golubovskaya [1,*]

Supplementary Materials

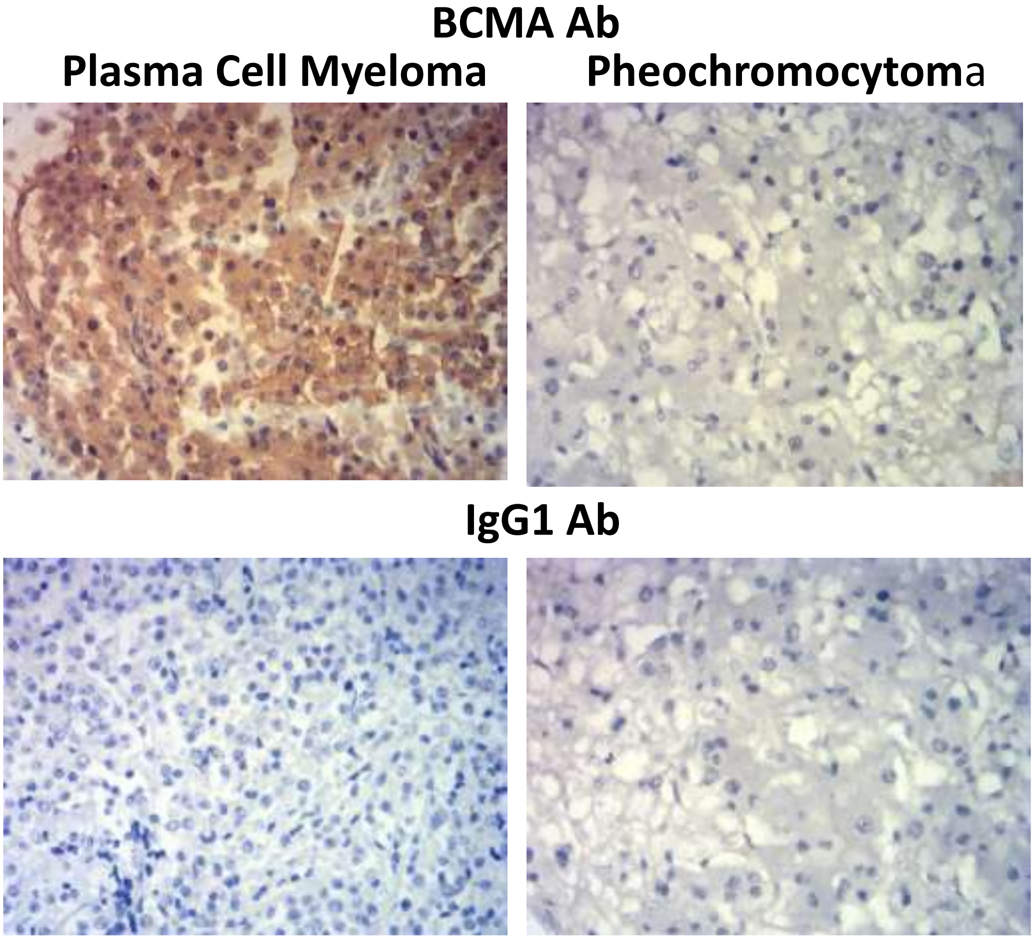

Figure S1. The IHC staining with BCMA Ab 4C8A and isotype control (1:300 dilution) was performed on primary bone marrow myeloma tissue sample and negative control adrenal gland pheochromocytoma tissue. 400 × magnification.

© 2018 by the authors. Submitted for possible open access publication under the terms and conditions of the Creative Commons Attribution (CC BY) license (http://creativecommons.org/licenses/by/4.0/).
